# Prof. Dr. M. Iqbal Choudhary-A lifetime career dedicated to remarkable services in “natural products sciences”

**DOI:** 10.3389/fphar.2023.1119419

**Published:** 2023-01-26

**Authors:** Hidayat Hussain, Hina Siddiqui, Ioannis P. Gerothanassis

**Affiliations:** ^1^ Department of Bioorganic Chemistry, Leibniz Institute of Plant Biochemistry, Halle (Saale), Germany; ^2^ H.E.J. Research Institute of Chemistry, International Center for Chemical and Biological Sciences, University of Karachi, Karachi, Pakistan; ^3^ Section of Organic Chemistry and Biochemistry, Department of Chemistry, University of Ioannina, Ioannina, GR, Greece

**Keywords:** M. Iqbal Choudhary, scientist, scientific achievements, drug discovery, bioorganic, medicinal chemistry

## Short biography

It is a great honor and a pleasure for us to serve as the Guest Editors for this especial issue of “*Frontier in Pharmacology*” dedicated to Prof. Dr. Muhammad Iqbal Choudhary on his pioneering contributions in the field of Bioorganic, Synthetic, and Natural Product Chemistry. This special collection, honoring Prof. Dr. M. Iqbal Choudhary on his immense scientific contributions, and S and T (Science and Technology) capacity building services, represents an excellent opportunity to celebrate a remarkable chemist.


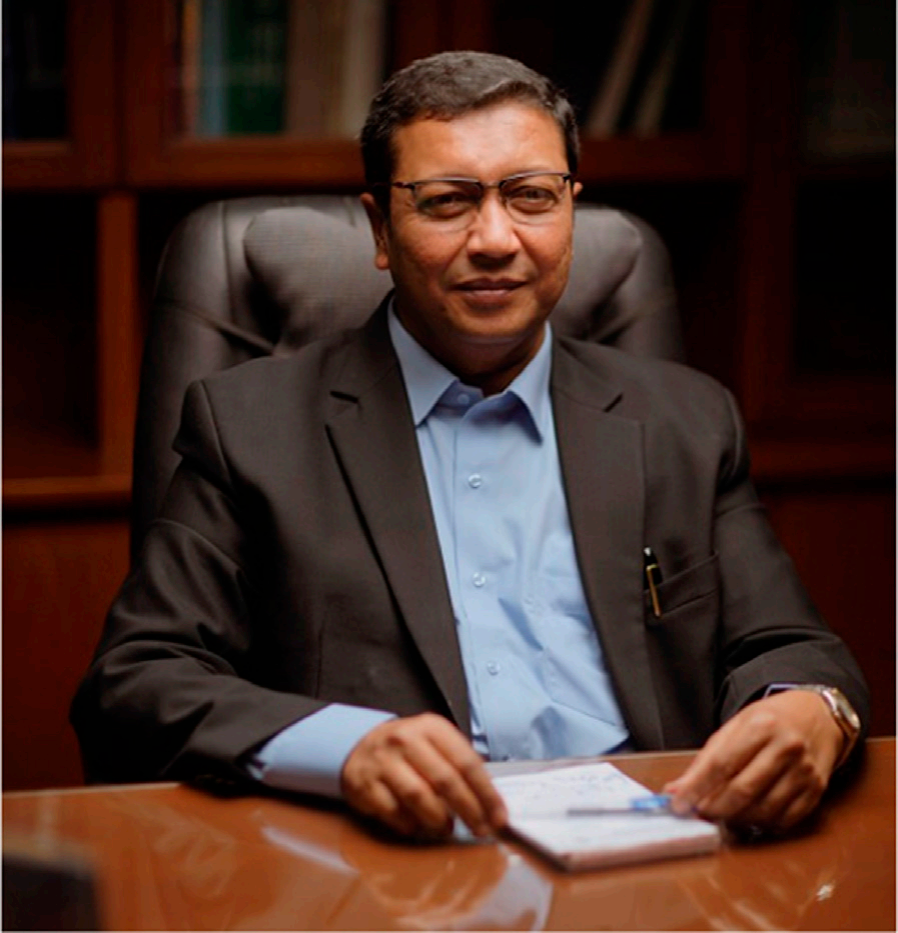


Prof. Dr. M. Iqbal Choudhary was born on 11 September 1959 in Karachi, Pakistan and completed his undergraduate studies at the University of Karachi. He completed his Ph. D. degree under the supervision of Prof. Dr. Atta-ur-Rahman FRS, a renowned natural product chemist, at the H. E. J. Research Institute of Chemistry, University of Karachi in 1987. He completed his doctoral research work from the Pennsylvania State University, United States during 1985–86. Later he did his post-doctoral studies at the Department of Chemistry, Cornell University and Department of Medicinal Chemistry, Purdue University, United States. He was also a Senior Fulbright Fellow at the Scripps Institute of Oceanography, University of California at San Diego, United States.

## Career and scientific achievements

Prof. Dr. M. Iqbal Choudhary now serves as a Distinguished National and Meritorious Professor, Director at the International Center for Chemical and Biological Sciences (ICCBS), H. E. J. Research Institute of Chemistry (www.iccs.edu), and Dr. Panjwani Center for Molecular Medicine and Drug Research), University of Karachi, Karachi, Pakistan. In addition, he is also serving as the Coordinator General COMSTECH [Organization of Islamic Cooperation (OIC) Ministerial Standing Committee on Scientific and Technological Cooperation] (www.comstech.org), and the Vice President of Central and South Asia of the World Academy of Sciences (TWAS). He is among the most leading scientists of Pakistan and he is also the author of over 1,266 research publications in reputed international journals, as well as 64 US patents. Notably, the cumulative impact factor of his publications is over 2,550 (calculated in Dec. 2022) and his citations exceeding 36,700 (h-Index: 80). Under his direct supervision, over 100 Ph. D. students have graduated. In addition to this, several hundred foreign students, including women students’ researchers, from Nigeria, Nepal, Bangladesh, Sri Lanka, Iran, Kazakhstan, Sudan, Cameroon, Ethiopia, Mauritius, Jordan, Lebanon, Indonesia, China, Burkina Faso, and Egypt, have been trained for their doctoral and post doctoral studies at his laboratories. He has served as a visiting faculty member in many prestigious universities all over the world, including Purdue University, Cornell University, Scripps Institution of Oceanography, Pennsylvania State University, University Rhode Island, and various top Universities in the China, United Kingdom, Malaysia, Saudi Arabia, Kazakhstan, and Iran.

Prof. Choudhary has written or edited 94 books, including “Solving Problems with NMR Spectroscopy, Edition 2” (Academic Press (Elsevier), USA in the years 2006 and 2015) and “Bioassay Techniques for Drug Development” (Harwood Academic Publishers, Amsterdam) (1999). Currently, he is the editor of many other prestigious international book series and several international science journals, including “*Mini Reviews in Medicinal Chemistry*”, and “*Current Bioactive Compounds”* and the rest.

## Honors and awards

Prof. Choudhary has been awarded several prestigious national and international awards, including the Friendship Award by the Chinese Government (2022), Gold Medal conferred by the International Turkic Academy (ITA), Kazakhstan 2022; Mustafa (PBUH) Prize and Award (Laureate) in Bioorganic Chemistry (Mustafa PBUH Science and Technology Foundation) 2021; Appointed by UNESCO as a UNESCO Chair holder on Medicinal and Bio-Organic Natural Product Chemistry (2020); President’s International Fellowship Initiative (PIFI), “Distinguished Scientist Award” Chinese Academy of Sciences (2020); Doctor of Science (D.Sc.) from Al-Farabi Kazakh National University, Kazakhstan (2019) and University of Karachi (2005); COMSTECH Award in Chemistry (2010); Civil Award *Hilal-e-Imtiaz* by the President of Pakistan (2007); Civil Award *Sitara-e-Imtiaz* by the President of Pakistan (2001); First Khwarizmi International Award and Prize from the President of the Islamic Republic of Iran (2006); Economic Cooperation Organization (ECO) Excellence Awards in Education 2006 from the President of Azerbaijan; Distinguished National Professor by the Higher Education Commission (2004); the Academy of Sciences for the Developing World (TWAS) (2003); Civil Award *Tamgha-i-Imtiaz* by the President of Pakistan (1999); Prof. Riazuddin Siddiqui Gold Medal of Pakistan Academy of Sciences (1992); The Third World Academy of Science (Trieste, Italy) Young Scientists Award (1994); National Book Foundation Prize for the Best Science Book (1995); and Fellow, Prof. Abdussalam (Nobel Laureate) Prize in Chemistry (1989).

Prof. Choudhary was a Visiting Professor at several international universities, including the University of Rhode Island, USA; Manchester Metropolitan University, United Kingdom; King Saud University; King Abdulaziz University, Saudi Arabia; Al-Farabi Kazakh National University, Kazakhstan, University Malaysia Pahang, Malaysia; National Pharmaceutical Chemistry of Human University of Chinese Medicine, China; Guangxi Normal University and State Key Laboratory for Chemistry and Molecular Engineering of Medicine Resources, China; T.C.M. and Hospital of Southwest Medical University Luzhou, Sichuan, China.

## Key contributions

### Establishment of higher education institutions

Prof. Choudhary is currently leading two large institutions, the ICCBS (A UNESCO Cat. II center), and the COMSTECH. Under the dynamic leadership of Prof. Dr. Choudhary the ICCBS has emerged as one of the finest academic and research institutions in the developing world. He has led the major expansion of this premier research institution since 2002, both in terms of infrastructure and academic excellence. The institute received the prestigious IsDB (Islamic Development Bank) Prize twice (2004 and 2010) for the best science institution in the OIC region and selected as an UNESCO Category II Institution, and WHO Collaborating Center. As the Director of the ICCBS, Dr. Choudhary has opened the doors of state-of-the-art facilities for researchers, not only from Pakistan but also from other countries.

Prof. Choudhary has trained many young scientists from developing countries and helped them to initiate and continue their research despite the constrained environment in their countries. A large number of these young scholars have received their Ph. D. degrees based on their research work conducted under the supervision of Dr. Choudhary at the ICCBS. These scholars have developed into well-established scientists, serving their nations. Over 400 scientists (since 2004) from developing countries have been trained, which also resulted in hundreds of joint international publications. Dr. Choudhary facilitated the scientific visits of women scholars from developing countries to the ICCBS and other institutions, which have resulted in capacity building and continuity in their scientific career.

### Key contributions in drug discovery

Prof. Choudhary has skillfully employed his deep knowledge of chemical principles along with biological functions in the discovery of a numerous intriguing compounds with significant therapeutic potentials (Siddiqui et al., 2020). Notably, Prof. Choudhary research group has discovered and established new clinically important enzyme inhibitors, which can be employed to stop the biological functions involved in enzyme-related disorders. His esteemed group have also discovered various new classes of lead compounds including numerous novel natural products and synthetic compounds, which possessed significant acetylcholinesterase, urease, α-glucosidase, and aromatase inhibitors. Some of these chemical entities are currently in different phases of human clinical and preclinical trials.

Dr. Choudhary and his research team have worked extensively on the plants used by the local population for the treatment of epilepsy and other neurodegenerative disorders (Shaheen et al., 2021). He along with his team has discovered isooxylitones A and B, the two most active antiepileptic secondary metabolites and patented in the United States. Interestingly these compounds were isolated from *Delphinium denudatum* and is currently in phase 2 clinical trials. These results have been published in many international journals and patented in the United States.

Moreover, he has developed an effective nutraceutical formulation for Parkinson’s disease, which has been patented and is currently in phase-II human clinical trials. Discovery of anti-leishmanial compounds is another field of his interest (Atta-ur-Rahman et al., 2012; Choudhary et al., 2005; Samdani et al., 2012). His team has successfully conducted multicenter clinical trials on the most potent compound and/or extracts from *Allium sativum* L., and *Physalis minima* L. Prof. Choudhary has identified drug candidates for the treatment of breast cancer which are several fold more active than existing clinical drugs (Choudhary et al., 2020). These drug candidates are currently in preclinical trials.

Interestingly, Prof. Choudhary has made pioneering contributions on the development of chemical entities which can reverse multidrug resistance (MDR) in *Staphylococcus aureus,* and *Pseudomonas aeruginosa*. Furthermore, Prof. Choudhary has discovered various enzyme inhibitors against various critical drug targets (enzymes involved in cancer, diabetes, peptic ulcer, and Alzheimer’s disease) and antiglycating agents from natural sources, synthetic compounds, and bio-transformed metabolites.

## Global S and T (science and technology) capacity building

On the platform of COMSTECH (www.comstech.org), Prof. Choudhary is responsible for S&T cooperation and capacity building in member states in four continents. Since his appointment as the Coordinator General of COMSTECH, OIC-COMSTECH (Standing Committee of 57 Ministers of OIC member countries) has embarked on a journey of making international joint ventures, and fund raising for science and technology capacity building in largely LDCs (L*east Developed Countries*). This includes OIC’s largest research mobility program, COMSTECH Consortium of Excellence (CCoE), COMSTECH-Africa Program in Health and STI in Niger, Uganda, Burkina Faso, Gambia, Nigeria, and Chad, Emerging Technology Initiative with IsDB and ICCI&A, Science in Exile Program (TWAS and UNESCO), Distinguished Scholars Program, OIC Technology and Innovation Portal, Research Fellowships in the fields of “Virology and Vaccine Development” with ICGEB, Women in Science Program with Women Development Organization (WDO), Technician Training Program for Capacity Building, and the Joint Research Grants Program for Young Scientists with IFS (International Foundation For Science). Besides this, under his mentorship, various technology exhibitions related to healthcare solutions, artificial intelligence, and information technology had been organized to link universities with the health industry for providing technology integrated solutions of patients.

Prof. Choudhary has helped in the establishment and strengthening of institutions, research centers, and laboratories in various countries of the developing world, including;

• Turkey: “Natural Product and Pharmacognosy Laboratory”, Gazi University, Ankara.
• Nigeria: “Iqbal Choudhary Center for Natural Product Research”, Benin, Nigeria
• Cameroon: “Natural Product Chemistry Center”, University of Younde’ I.
• Sudan: “Medicinal Plants Research Institute (MAPRI)”, National Research Center, Khartoum.
• Bangladesh: “Laboratory for Research on Anti-Diabetic Plants”, Dhaka.
• Kazakhstan: “Medicinal Plants Research Institute”, Al-Farabi National Kazakh University.
• Sri Lanka: “Center for Advanced Research”, Sir General Kotelawala Defense University, Colombo.
• Iran: “Natural Products Research Center”, Shaheed Bheshti University of Medical Sciences, Tehran.
• Saudi Arabia: “Laboratory for Bioorganic Chemistry”, King Abdulaziz University, Jeddah.

**FIGURE 1:**
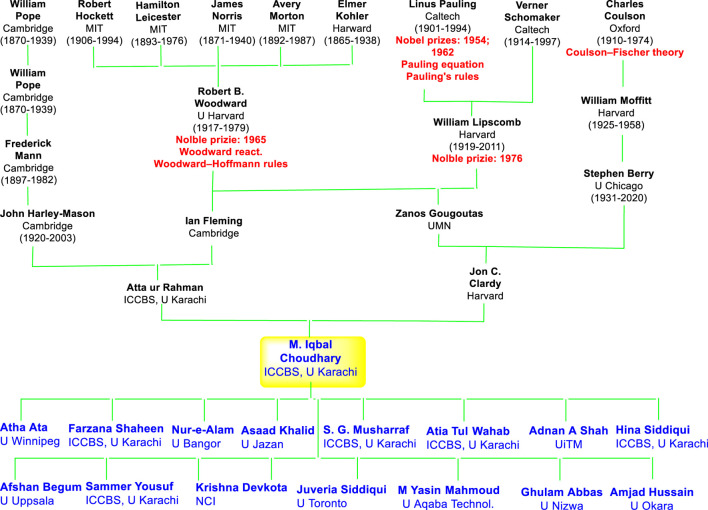
Muhammad Iqbal Choudhary’s academic family tree; only some of the Ph.D. students he has supervised and who eventually became university professors or researcher are included; all researchers including Ph.D. students) associated with him would exceed the limits of this compilation.

Prof. Choudhary has always been a highly motivated, innovative, and hardworking researcher with gifted skills of a keen observer. As a mentor he has been a very encouraging and stimulating mentor to his students. He has this unique ability to teach students in such a way that they can visualize the concept in their mind easily, which reflects his command of the subject. He discusses science with reasoning and logic which has enabled him to supervise a large number of scholars and in this way creating a highly competitive, international and interdisciplinary working environment. Many of his former national and international students are now working in top institutions in Pakistan and the world as Professors and researchers (Figure 1). Indeed, his passion for science, work, and fascinating scientific ideas has inspired entire generations of scientists around the globe, as showed by the contributions to this Special Collection.
